# A quality indicator set for use in rehabilitation team care of people with rheumatic and musculoskeletal diseases; development and pilot testing

**DOI:** 10.1186/s12913-019-4091-4

**Published:** 2019-04-29

**Authors:** Inger Johansen, Mari Klokkerud, Audny Anke, Janne-Birgitte Børke, Thomas Glott, Uno Hauglie, Audhild Høyem, Atle Klovning, Karin Anna Lande, Mona Larsen, Jan Egil Nordvik, Sigrid H. Wigers, Irene Øyeflaten, Kaare Birger Hagen, Ingvild Kjeken

**Affiliations:** 1 National Advisory Unit on Rehabilitation in Rheumatology, Diakonhjemmet Hospital, P.O. Box 23, 0319 Oslo, Norway; 2 Department of General Practice, Institute of Health and Society, Faculty of Medicine, University of Oslo, P.O. Box 1130 Blindern, 0318 Oslo, Norway; 3 Department of Rehabilitation, University Hospital of North Norway, Tromsø, Norway; 4 Faculty of Health Sciences, Department of Clinical Medicine, UiT The Arctic University of Norway, Tromsø, Norway; 5 National Advisory Unit on Occupational Rehabilitation, Haddlandsvegen 20. 3864, Rauland, Norway; 6 Uni Research Health, Nygårdsgaten 112, 5008 Bergen, Norway; 7 Unicare Jeløy Rehabilitation Centre, Bråtengaten 94, N-1515 Moss, Norway; 8 Norwegian Rheumatism Association, PO Box 2653, Solli, N-0203 Oslo, Norway; 9 Sunnaas Rehabilitation Hospital, 1453 Bjørnemyr, Norway; 10 Department of Physical Medicine and Rehabilitation, St. Olavs Hospital, Trondheim University Hospital, Trondheim, Norway; 11 The Municipality of Sandefjord, PO Box 2025, 3202 Sandefjord, Norway; 12 Rehabilitering Vest AS, PO Box 2175, 5504 Haugesund, Norway; 13 Centre for Quality Improvement and Development, University Hospital of North Norway, Box 20, N-9038 Tromsø, Norway

**Keywords:** Rehabilitation, Musculoskeletal diseases, Quality indicators, Health care, Delphi technique

## Abstract

**Background:**

Systems for monitoring effectiveness and quality of rehabilitation services across health care levels are needed. The purpose of this study was to develop and pilot test a quality indicator set for rehabilitation of rheumatic and musculoskeletal diseases.

**Methods:**

The set was developed according to the Rand/UCLA Appropriateness Method, which integrates evidence review, in-person multidisciplinary expert panel meetings and repeated anonymous ratings for consensus building. The quality indicators were pilot-tested for overall face validity and feasibility in 15 specialist and 14 primary care rehabilitation units. Pass rates (percentages of “yes”) of the indicators were recorded in telephone interviews with 29 unit managers (structure indicators), and 164 patients (process and outcome indicators). Time use and participants’ numeric rating of face validity (0–10, 10 = high validity) were recorded.

**Results:**

Nineteen structure, 12 process and five outcome indicators were developed and piloted. Mean (range) sum pass rates for the structure, process and outcome indicators were 59%(84%), 66%(100%) and 84%(100%), respectively. Mean (range) face validity score for managers/patients was 8.3 (8)/7.9 (9), and mean answering time was 6.0/5.5 min. The final indicator set consists of 19 structure, 11 process and three outcome indicators.

**Conclusion:**

To our knowledge this is the first quality indicator set developed for rehabilitation of rheumatic and musculoskeletal diseases. Good overall face validity and a feasible format indicate a set suitable for monitoring quality in rehabilitation. The variation in pass rates between centers indicates a potential for quality improvement in rheumatic and musculoskeletal rehabilitation in Norway.

**Electronic supplementary material:**

The online version of this article (10.1186/s12913-019-4091-4) contains supplementary material, which is available to authorized users.

## Background

Rheumatic and musculoskeletal diseases (RMDs) are among the most regular disorders in the general population and have a large economic and social impact on societies [1]. Due to increased life expectancy and overweight, the number of people with RMDs will rise in the coming decades [2]. Even though biological medicines have revolutionized the treatment of inflammatory rheumatic diseases in the past one-and-a-half decades, pharmacological treatment of the majority of RMDs still mainly targets alleviation of symptoms [3, 4]. Successful management of these diseases therefore is strongly dependent on the patient’s ability to implement life-style changes and self-management strategies, often introduced through rehabilitation programmes. Several studies have shown that patients with RMDs benefit from rehabilitation, but the effect seems to decline over time, and most people are back to their initial health status six to twelve months after discharge [5–7].

When it comes to the organization of rehabilitation in Norway, healthcare statutes push for a shift in the direction of shorter in-patient stays or day programmes in specialized care, and more rehabilitation and better follow-up in primary care. However, several recent reports conclude that the quality of rehabilitation services in general is low, characterized by a lack of coordination across levels of care, and with divergences in content and quality, especially in primary care. Furthermore, the same reports underline that systems for monitoring effectiveness and quality across health care levels are needed [8, 9]. One method to monitor the quality of patient care and to clarify areas of improvement is to develop quality indicators (QIs) of care [10]. QIs can be defined as “measurable elements of practice performance for which there is evidence or consensus that it can be used to assess the quality, and hence to change the quality, of care provided” [11]. The purpose of healthcare quality indicators is to serve as i) a basis for management for authorities and owners, ii) tools for internal quality improvement and iii) support for patients in choosing service providers [10]. QIs are related to structure (the settings in which care occurs), process (what is actually done in giving and receiving care), or outcome of health care [12].

QI sets to manage different rheumatic diseases have been developed [13–18]. Some of the QIs are adapted to a specific diagnosis, others are more generic. However, most quality sets mainly include process indicators, some of them related to rehabilitation and some not, and the development processes were suboptimal [15, 18]. Comprehensive and resource demanding quality assurance and accreditation systems for social and rehabilitation services exist [19–21], as do human rights based indicator framework systems to assess countries´ efforts to strengthen rehabilitation provision and policy [22]. Nevertheless, to our knowledge, no valid and feasible indicator set has been developed for use in rehabilitation of RMDs, covering both structure, process and outcome quality and using appropriate methodology.

The purpose of this project was to develop and pilot test a set of QIs for use in rehabilitation team care of people with RMDs.

## Methods

We used a design based on the Rand/UCLA Appropriateness Method, which has been widely used to develop healthcare QIs [23]. The method integrates evidence review, in-person multidisciplinary expert panel meetings and repeated anonymous ratings for consensus building [23, 24], and was conducted in four separate steps: i) preparatory phase, ii) consensus building, iii) pilot testing, and iv) consensus of the final QI set.

### Preparatory phase

#### Definitions and criteria

A broad national working group of nine members was established, approved by The Norwegian Health Directorate. The sampling strategy was non-random, aiming for a broad and relevant composition for the project. Both genders (three men, six women) and different geographic areas of Norway were represented. The professions were physician, nurse, physical- and occupational therapist (two of each), representing clinicians, researchers and administrators. The Norwegian Rheumatism Association nominated a patient representative. Four of the working group members later became participants of the broader expert panel.

The working group prepared the consensus process in an initial meeting. Rehabilitation was defined according to the definition provided by the Norwegian Ministry of Health and Social Affairs as “… a process, or set of processes, which is (are) planned and limited in time, where several professions or services cooperate in assisting the individual user in his or her own efforts to achieve best possible functioning and coping capabilities, and promoting independence and participation in society” [25]. Further, rehabilitation was understood as a process or trajectory that reaches across levels of care. The core professions in rehabilitation in primary care rehabilitation were agreed to be physical therapist(s), occupational therapist(s), nurse(s) and physician(s), and in specialist care, at least one more profession in addition to these.

#### Systematic search for QIs in rehabilitation

We conducted a systematic literature search in Medline, Embase, Amed, PsycINFO, PubMed and Cochrane from January 1980 to October 2014 for existing QIs in rehabilitation. Search terms were Quality Indicators, Health Care, Structure or Process or Outcome, Quality and Rehabilitation. The search was not limited to diagnostic groups or study design. We included only publications available in English, German, or Scandinavian languages. A total of 660 publications were identified, of which 12 described QIs for rehabilitation. These concerned rehabilitation in general (*n* = 4), rheumatoid arthritis or osteoarthritis (*n* = 5), and low back pain, osteoporosis or older people (1 publication each). We found no publications describing a QI set for rehabilitation of RMDs. However, several main themes for quality measurement were identified. This informed the process of generating a set of proposed quality domains, which later in the process were operationalized into specific QIs. The literature search was updated in March 2019. Eighty-one new publications were identified, of which three described QIs or quality measures for different managements of rheumatoid arthritis and osteoarthritis [16], psoriatic arthritis [17] and inflammatory arthritis [18]. One paper described human rights based indicator framework systems to assess countries´ efforts to strengthen rehabilitation provision and policy [22].

#### The expert panel

A broad national expert panel comprising 18 members was established (Table 1) [23]. To be deemed an expert, she/he had to have profound clinical, research and/or administrative experience and knowledge and be seen as a trusted expert in the field of rehabilitation. The sampling strategy was non-random selection aiming to seek participants from different geographical areas, levels of care, and with different professional training, position and rehabilitation experience. The patient representatives in the panel had rheumatoid arthritis and low-back pain and were nominated by the Norwegian Rheumatism Association and the Norwegian Back Association.

**Table 1:** Characteristics of members in the expert panel

Profession	Gender		Health care level		Professional role^a^			Rehabilitation experience		
	F^b^	M^c^	S^d^	P^e^	Clinician	Researcher	Admin	RMDs	Vocational	General
Patient representative	1	1						2		
Physical therapist	4	1	3	2	3	2	2	2	1	2
Occupational therapist	3	1	2	2	2	1	3	1		3
Nurse	1		1	1	1		1			2
Social educator	1						1			
Physician	3	1	4		3	3	4	3		3
Psychologist		1	1			1	1			1

^a^Most members had more than one professional role. ^b^Female. ^c^Male. ^d^Specialist care. ^e^Primary care

### Consensus building

In an introduction meeting, the expert panel was informed about the aims and frames of the project, results of the literature search and proposed quality domains, and was invited to propose additional domains. Thereafter, a second literature search was performed, related to empirical evidence for the proposed domains. The search terms were different synonyms of the proposed domains: “population selection criteria”; “discharge criteria”; “length of stay”; “bio-psycho-social assessment”; “patient participation”; “patient-centred”; “arena”; “facilities”; “equipment”; “systematic and targeted rehabilitation”; “staffing”; “multidisciplinary competence”, and “outcome”. All searches were combined with different search terms for rehabilitation; in addition, the search for outcome was combined with the search terms “patient satisfaction”, “rehabilitation outcome” or “clinical outcome” or “treatment outcome”. The searches were not limited to any diagnostic group.

Publications related to empirical evidence for the proposed domains were included in a reference list along with all relevant reports and recommendations. Subsequently, we prepared one fact sheet for each proposed quality domain including the rationale, definition and categorization of the domain, history and evidence with references to relevant literature, relevant patient populations and health care levels. Based on the reference list, the fact sheets and their own expertise, the panel members, in two voting rounds, reached a consensus on a set of quality domains.

#### Round 1

The fact sheets describing each quality domain and the reference list were distributed to all panel members by e-mail. Members anonymously rated each domain according to the following OECD criteria in the Health Care Quality Project [26]: Importance of what is being measured, scientific soundness of the measure (validity), usefulness (three sub-scales: *i* susceptibility of being influenced by the health care system, *ii* the degree to which the use of the quality measure results in desired outcomes, *iii* free from errors), and feasibility. Each criterion was scored on a Likert scale from 1 to 9 (9 = high degree of agreement).

#### Selection criteria

For each quality domain, medians of the nine point Likert scores were calculated and the domains were classified into three levels of appropriateness: 1) “appropriate” was defined as a panel median of 7–9 for all criteria. 2) “uncertain” (and included in the next voting round) was defined as a panel median of ≥6 in up to two criteria and a median of ≥7 in the rest, and 3) “inappropriate” (and excluded from further voting rounds) was defined as a panel median lower than the minimum scores for “uncertain”.

#### Round 2

The expert panel met for a second one-day, in-person meeting. The results from round 1 were discussed, and the panel decided to revise the wording and elements of some of the domains. Following this, the domains were assessed a second time, during which the panel members voted on the domains that had been rated “uncertain” in round 1.

After round 2, the results from the consensus process were presented to the expert group and discussed in a third one-day, in-person meeting. Each appropriate and uncertain quality domain was formulated as one or more specific QIs, and the panel reached consensus on a set of QIs to be pilot tested.

### Pilot testing

The purpose of the pilot testing was to test the face validity (the degree to which the instrument appears to be an adequate reflection of the construct to be measured [27]) and the feasibility of the QI set. Preferably, QIs are to be extracted from existing data sources such as clinical registers or patient records [26]. However, extracting data from the electronic patient records is not yet possible in Norway, and there is only one relevant patient register that covers a limited segment of the RMD population [28]. Therefore, two yes/no questionnaires were developed for the pilot testing; one for rehabilitation unit managers (addressing structure QIs) and one for patients (addressing process and outcome QIs). Several of the structure and process QIs were matched, to be able to align quality assessment from both a system and user perspective. One example of this might be to ask managers if the unit had written procedures in daily use for making an individual rehabilitation plan (structure QI) and to ask the patient if she had received such a plan (process QI).

Rehabilitation units from both specialist and primary care were invited to participate in the pilot testing, using a snowball method whereby expert panellists suggested units to be invited. Both oral and written information about the study was provided. The managers of the rehabilitation units answered the structure QIs in the beginning of the test period, whereas patients answered the process and outcome QIs one to two months after completion of a rehabilitation programme. The questionnaire was first sent by e-mail to the participants, who thereafter gave their answers in a telephone interview conducted by the first author (IJ) or a study secretary.

The time spent answering the questionnaire in the interview was recorded in minutes, and participants’ ratings of face validity were recorded on a numeric rating scale (0–10, 10 = to a large degree) according to the question: To what degree do you think the questions capture important elements in rehabilitation? The participants were also asked to comment on the content and comprehensibility of the questionnaire.

The study was approved by the Data Inspectorate/Data Protection Official (ref.no: 2015/16099). All participants provided written consent.

### Reaching consensus on the final QI set

Results from the pilot test were used as a basis for the discussion in the expert panel in a fourth meeting. Thereafter, the uncertain QIs were rated by the options *yes* or *no* for approval/disapproval of each QI in a final anonymous consensus round. The previously uncertain QI was included if 75% or more of the panel had approved it.

### Data analysis

Data were analysed in the statistical software SPSS version 21. Descriptive data were given in means and medians. QI pass rates (PRs) were calculated in percent. Separate PRs for each QI were calculated by dividing the number of participants passing the QI (reporting “yes”) by the total number of participants answering “yes” or “no” for the same QI. Correspondingly, summary PRs were calculated by dividing the total number of QIs passed by each participant by the total number of eligible QIs. Correlations between PRs were analysed by Pearson’s r and comparisons of PRs between specialist and primary care by Independent Samples T-test or Mann-Whitney U Test. The statistical significant *p*-value was set at = < 0.05.

## Results

### Consensus building

#### Proposed quality domains

**Fig. 1:**
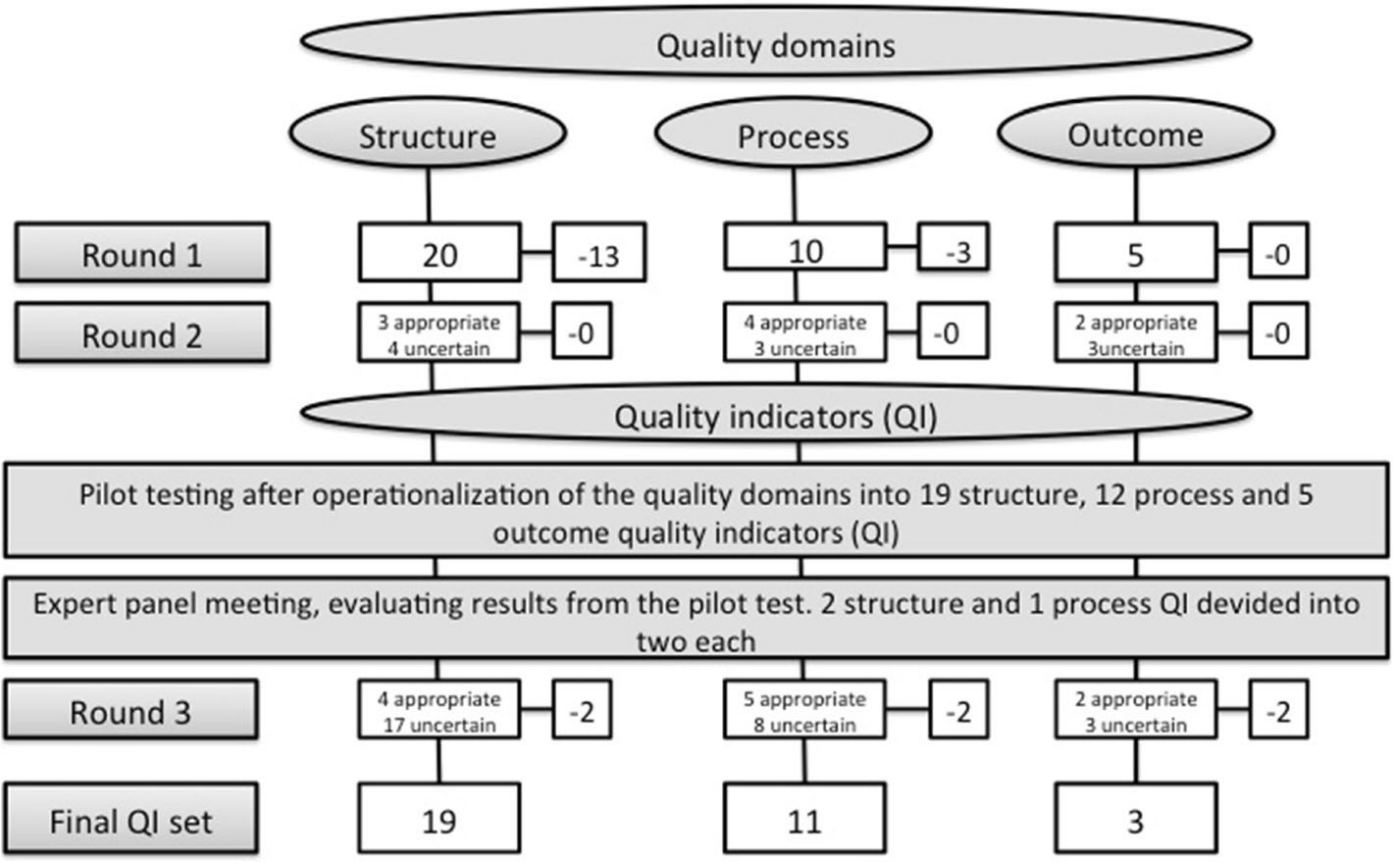
The consensus process and ratings of the quality domains and indicators

Based on assessment by experts, the initial literature review and relevant reports and recommendations, a first set of 35 quality domains was proposed (Table 2). Of these, 20 were related to structure, 10 to process and five to outcome (Table 2. Figure 1).

**Table 2:** Main themes of quality and corresponding quality domains. Results of the first two voting rounds

Main themes of quality and corresponding references	Proposed quality domains	Rating^e^	Cate-gory
Facilities and equipment [15] [25, 29–31]	Arena, universal design		S^a^
	Environment		
	focus on rehabilitation		S
	stimulating, supportive, accessible		S
	Equipment		
	appropriate and sufficient		S
	of high quality		S
Rehabilitation professionals; education, training and experience, skills, dedication and motivation, staffing ratio [15, 25, 29–32]	Minimum number of professions		S
	Access to professions outside ordinary staff		S
	Adequate staffing		P^b^
Admission an discharge criteria [15, 25, 31, 33]	Admission criteria		S
	Discharge criteria		S
	Defined patient target group	*	S
Systematic biopsychosocial assessment of the patient [15, 20, 25, 29–31, 34, 35]	Use of		
	validated assessment instruments	*	S
	instruments covering physical, mental and social aspects	*	P
	Multi professional assessment at		
	admission		S
	discharge		S
	Bio-psycho-social assessment at		
	admission		S
	discharge		S
	Initial bio-psycho-social assessment	**	P
The intervention; patient education, exercises, assistive devices, medication. User involvement in the rehabilitation process [15, 20, 25, 29–31, 34–36]	Procedure for patient participation in		
	planning and evaluating the	**	S
	rehabilitation process		
	goal setting	**	S
	Patient participated in		
	planning the intervention	**	P
	goal setting	**	P
	Procedure for		
	individual written rehabilitation plan	**	S
	regular team meetings with patients	*	S
	written individual plan for follow-up	*	S
	Patient received		
	a written rehabilitation plan	**	P
	a written individual plan for follow-up	*	P
	Weekly evaluation of the rehabilitation plan		P
	Team meetings with possibility for the patient, next of kin and external personnel to participate	*	P
	Length of rehabilitation stay		P
Health Related Quality of Life Restored or improved function in ADL Ability to return to pre-event dwelling Successful involvement in school, work, leisure and social activities [25, 29, 31, 34, 37, 38]	Improvement in HRQoL^d^	*	O^c^
	Improvement in function	**	O
	Goal attainment	**	O
	Satisfied with the rehabilitation	*	O
	Health complications or negative events	*	O

^a^Structure ^b^ Process ^c^ Outcome ^d^ Health Related Quality of Life ^e^ the quality domains are marked according to the results of the ratings, round 1 and 2: ** Appropriate * Uncertain. Not marked: Inappropriate

The literature searches related to the proposed quality domains identified 7866 publications related to “population selection criteria” (*n* = 1306), “discharge criteria” and “length of stay” (*n* = 900), “bio-psycho-social assessment” (*n* = 1482), “patient participation” and “patient-centred” (*n* = 979), “arena”, “facilities” and “equipment” (*n* = 823), “systematic and targeted rehabilitation” (*n* = 782), “staffing”, “multidisciplinary competence” (*n* = 974), and “outcome” (*n* = 620). The first author screened the titles and abstracts. A total of 38 articles were deemed relevant according to the quality domains and were included in the reference list, which served as background material for the consensus process.

#### Voting round 1 and 2

Fourteen expert panel members (78%) voted in round 1, and 17 (94%) in round 2. The rating rounds resulted in 19 quality domains to be included for pilot testing, seven structure, seven process and five outcome domains (Table 2. Figure 1).

### Pilot testing

The expert panel operationalized the quality domains into 19 structure, 12 process and five outcome QIs (Fig. 1).

A total of 29 rehabilitation units located in all regions of Norway agreed to participate in the pilot test; 15 from specialist care (10 rehabilitation institutions and five hospital departments) and 14 from primary care (five institutional, seven home based and two day units).

A total of 207 patients received the patient questionnaire, of whom 164 (79%) completed it. Forty-one did not answer repeated phone calls, and two were excluded due to cognitive impairment. In five of the units, no patients answered the questionnaire.

The majority of participants were female and admitted to rehabilitation at a specialist rehabilitation institution. They had one or several of the following diagnoses: inflammatory rheumatic disease (*n* = 115), osteoarthritis (*n* = 75), neck-, shoulder- and/or back-pain (*n* = 86), generalized musculoskeletal pain (*N* = 44), fractures (*N* = 36), and osteoporosis (*n* = 24). More than half were employed and had previously undergone rehabilitation. Compared to the participants in specialist care, participants in primary care were older and more predominantly lived alone. Other patient characteristics are shown in Table 3.

**Table 3:** Characteristics of 164 patients participating in pilot testing of the RMD rehabilitation QI set

	Total	Missing	Rehabilitation in		
			Specialist Care		Primary Care
			Hospital	Rehab institution	Munici- pality
N (%)	164/(100)	6(4)	33(20)	91(55)	34(21)
Female gender, N (%)	126 (77)	6	22 (67)	81 (89)	23 (68)
Age, mean(SD)	60 (16)	6	54 (14)	55 (12)	77 (13)
Living alone, N (%)	59 (36)	6	10 (30)	31 (34)	18 (53)
> 12 years of education, N (%)	80 (49)	10	19 (58)	47 (52)	14 (41)
Employment, N (%)	87 (53)	0	23 (70)	64 (70)	0 (0)
Previous rehabilitation, N (%)	86 (52)	8	23 (70)	43 (47)	20 (59)
One/more than one co-morbidity that could influence the rehabilitation process, N (%)	70 (43)/ 23 (14)	9/66	16 (48)/ 5(15)	38 (42)/ 15(16)	16 (47)/ 3 (9)

Mean time spent on answering the questionnaire was 6.0 min for managers and 5.5 min for patients, whereas mean (range) face validity scores were 8.3 (8) and 7.9 (9), respectively.

#### Pass rates (PRs)

**Fig. 2:**
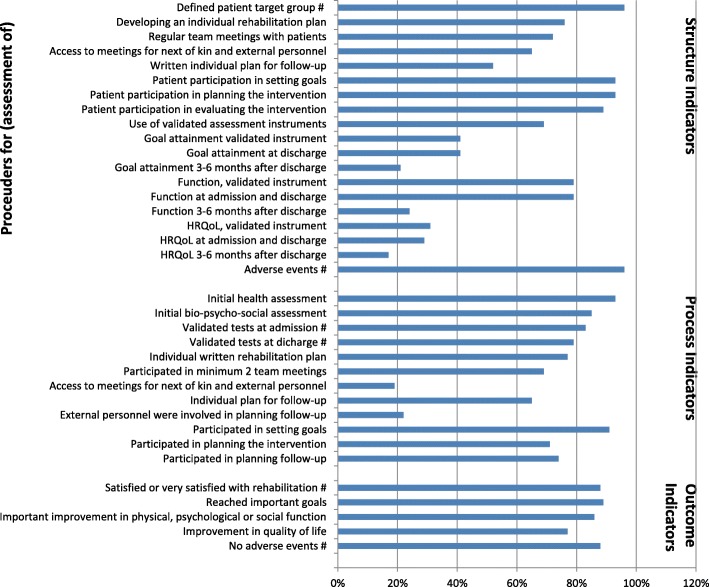
The QIs and corresponding PRs. The QIs rejected in round 3 are marked with #

Figure 2 shows the QIs and the corresponding PRs from the pilot test.

The PRs for structure QIs ranged from lowest 17% for the QI *Assessment of HRQoL 3–6 months after discharge* to highest 96% for the QIs *Defined patient target group* and *Assessment of adverse events.*

The PRs for process QIs ranged from lowest 19 and 22% for the QIs *Access to meetings for next of kin and external personnel* and *External personnel were involved in planning follow-up*, to highest 93% for the QI *Initial health assessment* and 91% for the QI *Participated in setting goals*.

The PRs for the five outcome QIs ranged from 77 to 89%.

**Fig. 3:**
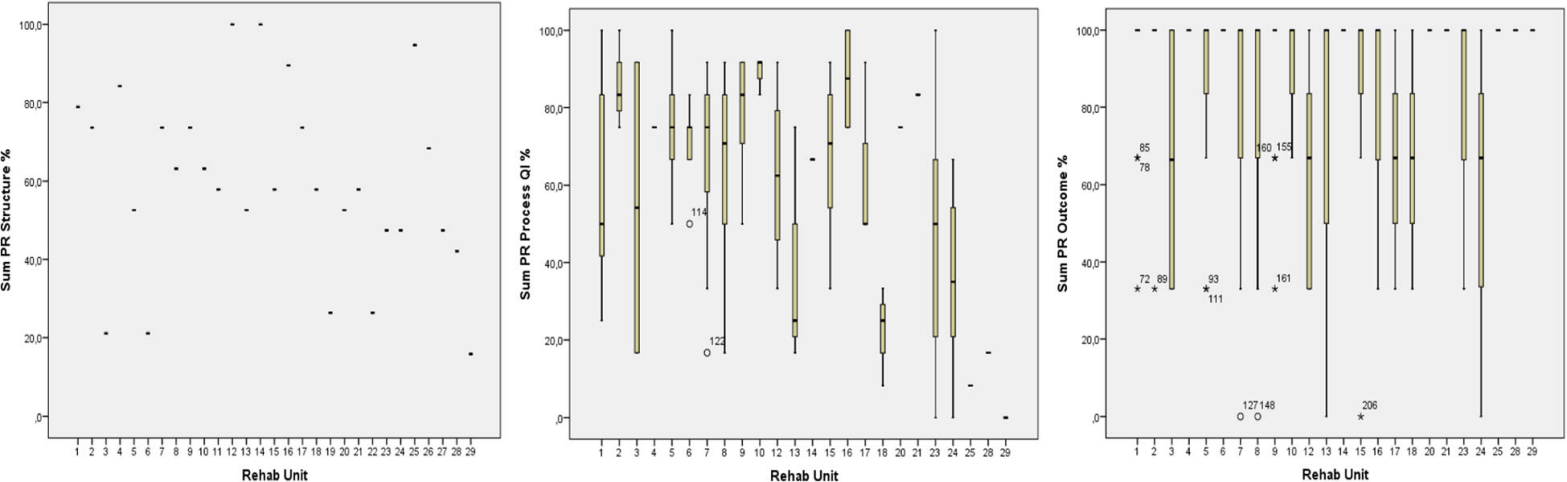
Variations in PRs visualized in a point diagram for the structure QIs, and boxplots for the process and outcome QIs. Specialist rehabilitation units are unit number 1, 2, 5, 6, 7, 8, 9, 10, 11, 12, 13, 16, 17, 18, and 21. The other units´ numbers are primary care

Figure 3 shows the variations in sum PRs for the QIs. Two of the rehabilitation units reached a 100% sum PR for the structure indicators, and four units reached ≤25%. 4% of the patients reached a 100% sum PR of the process indicators, and 11% reached ≤25%. A total of 70% of the patients passed all three outcome indicators *Improvement in quality of life, Important improvement in physical, psychological or social function* and *Reached important goals*. 13.8% passed one or no outcome indicator.

Results for rehabilitation unit number 11, 19, 22, 26 and 27 (Fig. 3) are not given in the process and outcome diagrams, since no patients in these units answered the questionnaire.

The mean (range) sum PRs for the structure, process and outcome QIs were 59%(84%), 66%(100%) and 84%(100%), respectively.

In general, the mean sum PRs were higher in specialist compared to primary care; for structure QIs 64.2 and 58.4% (*p* = 0.3), and for process QIs 70.0 and 50.0%, (*p* = 0.001), respectively. Regarding outcome QIs the mean sum score was 83.3% in specialist care and 80.0% in primary care (*p* = 0.5).

The total mean sum PRs of the process and outcome QIs correlated strongly and positively to the PR of the outcome indicator *Satisfied or very satisfied with the rehabilitation* (r = 0.56 *p* < 0.001). 75% of the patients who passed the outcome QI *Important improvement in function* also passed the outcome QI *Improvement in quality of life*.

Regarding comments to the questionnaires, three patients and two managers commented on comprehensibility of some of the QIs, and four patients said it was difficult to answer only “yes” or “no”.

### Reaching consensus on the final QI set

Evaluation of the QIs after the pilot test resulted in dividing three of the QIs to ensure accurate answers to each question (Fig. 1). This concerned the structure and process indicators *Access to meetings for next of kin and external personnel* and the structure indicator *Procedures for an individual plan for follow-up.*

The third round (voter participation 72%) resulted in exclusion of the structure indicators *Defined patient target group* and *Assessment of adverse events,* the process indicators *Validated tests at admission and at discharge,* and the outcome indicators *Satisfied or very satisfied with the rehabilitation* and *No adverse events*, all marked with # in Fig. 2.

An additional file shows the final approved QI set (see Additional file 1).

## Discussion

The primary aim of this project was to develop and pilot test a QI set for use in rehabilitation of people with RMDs. To ensure a transparent and scientific process, we adhered to the Rand/UCLA Appropriateness Method entailing that a broad expert group met several times and engaged in anonymous voting rounds.

The nineteen of the 35 proposed quality domains, which had been rated appropriate or uncertain in the voting rounds, were operationalized into 19 structure, 12 process and five outcome QIs formulated as yes/no questions. The pilot testing in 29 rehabilitation units and by 164 patients was followed up with an evaluation of the test results and a final voting round of the uncertain QIs in the expert panel, which resulted in 19 structure, 11 process and three outcome QIs.

When we updated the literature search, two new quality indicator sets and a systematic review of quality measures were detected [16–18]. Even though these sets to some degree align with the present, they are more pointed towards specific RMDs. Thus, to our knowledge, the present QI set is still the first one developed to measure quality in rehabilitation covering the whole RMD group and both structure, process and outcome quality and using appropriate methodology. Considering that RMDs have become the main contributor to disability in the Western world [1], assessing the quality of care will become increasingly important to policy-makers, providers, and patients.

The pilot revealed substantial potential for improvement in several important quality domains for many participating units. Three domains stood out. The first was *Evaluation meetings along the rehabilitation process with possibility to participate for the patient, next of kin and external personnel.* This domain was developed into both structure indicators (mean sum PR from 65 to 72%), and process indicators (mean sum PR from 18 to 70%). The domain represents a basic principle in rehabilitation [39]. Patient-participation and self-determination are crucial to the outcome and quality of rehabilitation [40]. However, our results support governmental reports documenting that patient-participation in rehabilitation is less than optimal [41]. The importance of family involvement is also well known [42]. Both the low PRs and the normative value of this domain indicate that it is an appropriate component of a QI set for rehabilitation.

The second domain was *Us*e *of validated assessment instruments at admission, discharge and 3–6 months after rehabilitation.* This domain was developed into ten structure indicators (mean sum PRs from 17 to 69%). Standardized assessment is the first step in, and a fundamental part of, a rehabilitation process [8, 43]. At the individual level, it is essential as a basis for setting rehabilitation goals, planning interventions, and monitoring and evaluating effects over time. On a group level, assessment is important for evaluating and comparing results of various rehabilitation programmes, to develop and improve programmes and to evaluate cost-effectiveness [44]. Again the low scores in the pilot and the normative value of the domain indicate that this is an appropriate component of a QI set for rehabilitation.

The third domain with a notable potential for improvement was *Individual planning of follow-up, involving the patient and external personnel.* This domain was developed into both a structure indicator (mean sum PR 52%), and process indicators (mean sum PRs from 25 to 65%). The lack of coordination and cooperation between rehabilitation programmes and levels of care has been documented for years as one of the weakest elements in rehabilitation [8, 45]*,* and there are unfortunately few signs of improvement. When discharged from rehabilitation, patients too often feel isolated and poorly prepared for living with disability in the municipality and know too little about where to turn to access the appropriate further health care [45]. These results underscore the importance of this quality domain and indicate that a shift is still needed in the field of rehabilitation, away from the acute-illness, curative model to one that acknowledges the long-term nature of a life with chronic disability.

Overall face validity of the QI set was tested with one question in the pilot study. However, during the anonymous ratings, panel members scored proposed items according to whether it was an obvious measure of quality, if it measured the intended item, and if it captured significant aspects of health and disease or important not medical conditions valued by the patients [23]. Thus, validation of each item was an inherent part of the consensus process. However, checking visits at participating centres, and controlling for effects of different patient characteristics is suggested for further validation of the QI set.

A limitation of the proposed QI set is that it is based on self-reports from rehabilitation unit leaders and users/patients and lacks data traditionally perceived as structural indicators. For practical application and information about the resource use of the health care, the proposed QIs should be linked to such data, i.e. national registers on work participation or medication, in future studies.

Regarding the feasibility of the QI set, the simple yes/no questions and the short time both managers and patients spent on answering them are in congruence with claims that data collection for quality measurement must be simple and not resource demanding [26]. However, while the participants in this pilot test answered the questionnaires by telephone, the intention is to manage the QIs as a self-reported questionnaire. This might influence both the answers given and the time to complete.

Recall bias may also have influenced the results, since the questions were answered one to two months after the rehabilitation period. On the other hand, these answers may be more reliable, as the first emotional reaction after the rehabilitation has come to some distance, and patients have got time to test their goals and self-management strategies in their home environment.

While the objective of the project was to develop a QI set for use in rehabilitation of patients with RMDs, the QI set appears generic and overriding and not limited to specific disease groups. This can be explained by the fact that good quality in rehabilitation encompasses change processes aimed at enhancing activity, participation and quality of life, which to a large extent are generic and independent of diagnostic group [46, 47]. The broad expertise in the consensus group and the fact that the systematic literature searches were not limited to RMDs may also have contributed to the perceived generic nature of the set. This may increase its feasibility, since rehabilitation takes place largely in units with mixed disease groups, especially in primary care.

Since the basic principles of rehabilitation to a large degree are the same across Europe and often independent of diagnostic group, it would be useful to try to reach international consensus for a generic QI set, which could be used to compare the quality of rehabilitation across countries. Further, diagnosis specific health care quality indicators, such as the eumusc.net sets for rheumatoid arthritis and osteoarthritis [13, 14], may be combined with the present set in studies focusing on specific diagnostic groups.

The healthcare level at which rehabilitation takes place varies between countries. A contemporary policy in many countries is to move responsibility for rehabilitation from specialist to primary care. To be able to monitor the quality of patient rehabilitation trajectories over time and compare the quality at different care levels, we aimed at developing a QI set that is valid and feasible both in a specialist and primary health care context. One potential risk of this strategy may be a ceiling effect in specialist care, since higher quality is expected at this level. Still, the results from the pilot test indicate considerable potentials for improvement at both levels.

The development of two separate questionnaires answered by rehabilitation managers and patients, respectively, may help to shed light on the black box of rehabilitation [48]. Analysing associations between structure, process and outcome indicators may identify factors of special importance to achieve positive outcomes. The matching of several of the structure and process indicators also allows for monitoring and comparing quality from both a system and user perspective, and for individual rehabilitation units as well as in a larger context. The pilot test showed clear differences in PRs for several of the matched indicators. However, the small scale testing does not justify for firm conclusions regarding these items.

A limitation in the pilot test was that the participating rehabilitation units were not randomly selected and were predominantly expected to give rehabilitation of high quality. This selection bias may have resulted in higher PRs than are representative for the average Norwegian rehabilitation unit. Furthermore, the present QI set was developed and pilot tested in a Norwegian context. As far as we know, the content of Norwegian rehabilitation of RMDs is largely comparable to other Northern European countries [30]. Nevertheless, little is known about the Norwegian level of quality in rehabilitation of RMDs compared to other countries. The pilot testing of the QI set was small scale with the mandatory uncertainties of such studies. The validity of the QI set should therefore be tested in other settings and larger samples. To explore the perceived generic nature of the QI set, it should also be examined in mixed diagnostic groups.

## Conclusion

A QI set for the rehabilitation of RMDs has been developed and pilot tested according to the Rand/UCLA Appropriateness Method. The results from the pilot test indicate that the QI set has good overall face validity and is feasible for monitoring quality in rehabilitation. The large variation in PRs suggests a potential for quality improvement in rehabilitation.

## Additional file

Additional file 1: The final approved QI set. (DOCX 25 kb)

## Abbreviations

PR: Pass rate
QI: Quality indicator
RMD: Rheumatic and Musculoskeletal Disease
