# Supplementary material for: A quality indicator set for use in rehabilitation team care of people with rheumatic and musculoskeletal diseases; development and pilot testing
Source: BMC Health Serv Res. 2019 Apr 29;19:265. doi: 10.1186/s12913-019-4091-4 (PMC6489243; doi:10.1186/s12913-019-4091-4)
Supplement: Supplementary file 1 — The final approved QI set. (DOCX 25 kb) [file 12913_2019_4091_MOESM1_ESM.docx]

**Quality indicators in rehabilitation**

**Questionnaire for the rehabilitation unit**

The following is included in the written procedures^*^ of the rehabilitation unit that are in daily use^**^: (Answer “No” if both requirements are not met))

1. The user/patient shall participate in setting rehabilitation goals.
2. The user/patient shall participate in planning his/her own rehabilitation process.
3. A template is used to prepare an individual rehabilitation plan for the user/patient.
4. The user/patient shall participate in evaluating his/her ongoing process.
   1. There are at least two meetings between the user/patient and the interdisciplinary team, or between the user/patient and a professional who represents the interdisciplinary team, and
   2. The user/patient is asked before meetings if he/she wants their next of kin to attend any of the meetings.
   3. The user/patient is asked before meetings if he/she wants some of the professionals he/she will relate to after the rehabilitation to attend any of the meetings. This may include a physiotherapist, general practitioner or a person from work if participating in vocational rehabilitation.
5. The rehabilitation unit uses reliable^***^ questionnaires and/or functional tests to assess physical, mental and/or social conditions.
   1. The user/patient shall participate in preparing a specified written follow-up plan (aside from the epicrisis) for the follow-up process after the rehabilitation period. This plan shall also include the user's/patient's own efforts to maintain or improve function/health.
   2. If there is a need for healthcare support after the rehabilitation period, the relevant personnel are to be informed about the plan or participate in the development of the follow-up plan.
6. The user´s/patient´s goal/goal attainment is to be assessed with a reliable instrument
   1. at the beginning of the rehabilitation period
   2. at the end of the rehabilitation period
   3. 3–6 months after the rehabilitation period
7. The user´s/patient´s function is to be registered using a reliable instrument
   1. at the start of rehabilitation
   2. at the end of the rehabilitation period
   3. 3–6 months after the rehabilitation period

___________________________________________________________________________________________________________

*Written procedures = written or digital documents, procedures, method descriptions and/or check lists

**In daily use = part of the routine daily clinical practice

***Reliable questionnaires and/or functional tests = quality assured/validated questionnaires and/or functional tests

1. The user´s/patient´s health-related quality of life is to be assessed using a reliable instrument
   1. at the beginning of the rehabilitation period
   2. at the end of the rehabilitation period
   3. 3-6 months after the rehabilitation period

**Guide to the questionnaire**

- The professional(s) (manager, responsible professional or both) who fill out the questionnaire must be knowledgeable about the *written procedures* of the rehabilitation unit and know whether they are *in daily use*
- The answer “Yes” requires both that the rehabilitation unit has a written procedure pertaining to the specific question asked, and that this procedure is easily accessible and in daily clinical use

**Quality indicators in rehabilitation**

**Questionnaire for users/patients**

Questions 1 to 3 concern assessment and evaluation of you as a patient during the rehabilitation period:

1. Were your **health condition** and **life situation** assessed during the first days of your rehabilitation period? (Answer ‘no’ if both aspects were not assessed.)

If you have answered yes to question number 1, go to question number 2.

If you have answered no to question number 1, go to question number 3.

1. Did the assessments include both a physical examination and questions about mental and social conditions, network, home situation and - if relevant – your work situation?
2. Was a written plan developed for the rehabilitation period (comprising your rehabilitation goals, what you should practise etc.)?

Questions 4 to 6 concern your participation in the rehabilitation process:

1. Were you actively involved in setting specific goals for the rehabilitation period (mentioned in question 3 above)?
2. Were you actively involved in preparing a specific written plan for the rehabilitation period (mentioned in question 3)?
   1. Did you participate in at least two meetings with the interdisciplinary team or a professional representing the team during which your goal(s) and goal attainment so far were discussed?
   2. Were you asked if you wanted your next of kin to attend any of the meetings?
   3. Were you asked if you wanted professionals you will relate to after the rehabilitation period to attend any of the meetings, such as a physiotherapist, your general practitioner, the labour and welfare administration (NAV) or a person from work, if you are participating in vocational rehabilitation?

Questions 7 to 8 concern planning of the period after rehabilitation:

1. Apart from the regular epicrisis, was a written plan developed for the period after rehabilitation, including what you were expected to work on yourself?

If you have answered ‘yes’ to question number 7, go to question number 8.

If you have answered ‘no’ to question number 7, go to question number 9.

- 1. Did you participate in developing the plan (question number 7)?
  2. As a part of this plan, were you consulted as to whether you needed follow-up from healthcare or vocational professionals (NAV) or other personnel after the rehabilitation period?

Questions 9 to 11 concern your outcome as a result of the rehabilitation:

1. As a result of the rehabilitation period, have you achieved one or several goals that are important to you?
2. As a result of the rehabilitation period, have you achieved an improvement in your physical, mental and/or social functioning that is important to you?
3. As a result of the rehabilitation, do you think your quality of life has improved?

Thank you very much for taking the time to answer these questions!

The questionnaires are developed by National Advisory Unit on Rehabilitation in Rheumatology [www.nkrr.no](http://www.nkrr.no)
